# Acting mechanism and clinical significance of hsa_circ_0005927 in the invasion and metastasis of gastric cancer

**DOI:** 10.7150/jca.96749

**Published:** 2024-05-30

**Authors:** Yongfu Shao, Xuan Yu, Meng Hu, Jianing Yan, Min Miao, Guoliang Ye, Junming Guo

**Affiliations:** 1 Health Science Center, Ningbo University, Ningbo 315211, China.; 2 Department of Gastroenterology, the First Affiliated Hospital of Ningbo University, Ningbo 315020, China.; 3 Institute of Digestive Disease of Ningbo University, Ningbo 315020, China.

**Keywords:** gastric cancer, hsa_circ_0005927, invasion, metastasis, biomarker, immune infiltration

## Abstract

**Background:** An increasing number of studies have demonstrated that differentially expressed circular RNAs (circRNAs) play critical roles in carcinogenesis. However, the biological function and clinical significance of hsa_circ_0005927 during gastric carcinogenesis remain unclear. The aim of this study was to investigate the acting mechanism and clinical significance of hsa_circ_0005927 in the invasion and metastasis of gastric cancer (GC).

**Methods:** Hsa_circ_0005927 was detected in GC tissues, plasma and gastric juice from patients with GC, and its correlations with clinicopathological parameters were investigated. Receiver operating characteristic curves, Kaplan-Meier survival curves and a prognostic nomogram model were generated to analyze the diagnostic and prognostic value. Real-time cell analyzer, plate colony formation, and Transwell migration and invasion assays were utilized to assess GC cell proliferation, migration and invasion, respectively. Nucleoplasmic separation was applied to determine the distribution of hsa_circ_0005927 in cells. TargetScan and miRanda software were used for target microRNA (miRNA) prediction. Transcriptome sequencing and bioinformatics analysis were performed to annotate the functions of hsa_circ_0005927 in gastric carcinogenesis and metastasis from an RNomic perspective. Key target genes and immune cell infiltrations were analysed.

**Results:** Hsa_circ_0005927 was found downregulated in high-grade intraepithelial neoplasia (HGIEN) tissues and GC tissues. Hsa_circ_0005927 levels in GC tissues were negatively correlated not only with lymphatic metastasis and distal metastasis but also with overall survival and disease-free survival. As a screening biomarker for GC, plasma hsa_circ_0005927 levels significantly increased in the early stages of GC, with a sensitivity and specificity of 52.38% and 76.19%, respectively. Hsa_circ_0005927 was mainly distributed in the cytoplasm, and structurally, it possesses multiple miRNA response elements (MREs) that interact with five miRNAs. A total of 421 downstream target genes of hsa_circ_0005927 were identified by transcriptome sequencing; and bioinformatics analysis suggested that these genes were involved mainly in the negative regulation of the T-cell apoptotic process, the interleukin-27-mediated signaling pathway, growth factor activity, guanylate cyclase activity, transcriptional misregulation in cancer, the cGMP-PKG signaling pathway, and the GnRH signaling pathway during gastric carcinogenesis and metastasis. GUCY1A2 and STK32A are key target genes significantly associated with immune infiltration.

**Conclusion:** Our study revealed that hsa_circ_0005927 is a new player related to the invasion and metastasis of GC and is a potential indicator for early GC screening.

## Introduction

Gastric cancer (GC) remains the fifth most prevalent type of cancer globally and the fourth most common cause of cancer-related mortality 1. Unrestricted cell proliferation and metastasis are the basic biological characteristics of GC 2. Although a combination of surgery, neoadjuvant chemoradiotherapy, molecular-targeted therapy, and immunotherapy is the main treatment and has increased the survival rate and improved the quality of life of GC patients 3, GC remains highly lethal due to recurrence and metastasis 4. Therefore, studying the key molecular mechanisms of GC metastasis has clinical significance in terms of improving the treatment and prognosis of GC.

CircRNAs, a class of endogenous circular RNA molecules with their 5′ and 3′ ends covalently linked, have been demonstrated to play crucial roles in the genesis and development of cancers 5. As a class of biomolecules with important regulatory functions, circRNAs can participate in gene expression regulation at the epigenetic, transcriptional, and posttranscriptional levels, affecting the proliferation, differentiation, invasion, and metastasis of cancer cells through a variety of mechanisms, such as acting as microRNA (miRNA) sponges, interacting with RNA-binding proteins, regulating gene transcription and alternative splicing, and being translated into proteins 6-9. More importantly, circRNAs are highly abundant and stable and exhibit tissue- and disease-specific expression, which provides novel perspectives for the treatment of malignancies 6.

Hsa_circ_0005927 comprises 379 nucleotides in its sequence. The host gene of hsa_circ_0005927 is voltage-dependent anion channel 3 (VDAC3) gene, which is located at chr8:42259305-42260979. In this study, we explored the acting mechanism and clinical significance of hsa_circ_0005927 in the invasion and metastasis of GC. The expression level changes, functions, and potential applications of hsa_circ_0005927 in gastric carcinogenesis were also verified. Our results suggest that hsa_circ_0005927 is a new player related to the invasion and metastasis of GC and is a potential indicator for early GC screening.

## Materials and methods

### Patients and clinical specimens

Specimens were collected at the First Affiliated Hospital of Ningbo University between 2013 and 2022. A total of 104 paired GC tissues and nontumorous tissues (5 cm away from the tumor) were collected from surgical patients. A total of 37 healthy gastric mucosa samples, 34 gastritis samples, and 27 paired gastric high-grade intraepithelial neoplasia (HGIEN) samples were obtained from biopsy specimens. Peripheral venous blood was obtained from 45 healthy volunteers and 46 early GC patients after a 12 h overnight fast. Gastric juice samples were obtained from 42 healthy volunteers, 30 gastric ulcer patients, 16 chronic atrophic gastritis patients, and 30 GC patients during gastroscopy. Tissues were immersed in RNA-fixer Reagent (Bioteke, Beijing, China), whereas plasma and gastric juice were separated into RNase-free centrifuge tubes (Axygen, Union, CA, USA) and then stored at -80°C until use.

Written informed consent was obtained from each patient. The Human Research Ethics Committee of Ningbo University and the ethics committee of the First Affiliated Hospital of Ningbo University approved every aspect of this study (IRB No. 20120303 and No. KY20220101).

### RNA extraction and qRT-PCR detection

Tissue RNA and gastric juice/plasma RNA were extracted using TRIzol and TRIzol LS reagents (Ambion, Carlsbad, CA, USA), respectively. Then, reverse transcription was performed to generate cDNA using the GoScript Reverse Transcription (RT) System (Promega, Madison, WI, USA). Quantitative real-time reverse transcription-polymerase chain reaction (RT-qPCR) was performed using GoTaq qPCR master mix (Promega) on the Mx3005P QPCR System (Stratagene, La Jolla, CA, USA). All operations followed the manual or manufacturer's instructions. Glyceraldehyde 3-phosphate dehydrogenase (GAPDH), a housekeeping gene, was used as the control. The primers for GAPDH and hsa_circ_0005927 were synthesized by Sangon Biotech (Shanghai, China). Their sequences were as follows: 5'-CATCCATAAACCTTGCTTGGA-3' (sense) and 5'-GGCCTTCAATTTCCCACTCT-3' (antisense) for hsa_circ_0005927; and 5'-ACCCACTCCTCCACCTTTGAC-3' (sense) and 5'-TGTTGCTGTAGCCAAATTCGTT-3' (antisense) for GAPDH. The Δ*C*_q_ method was used to calculate the levels of hsa_circ_0005927.

### Cells and culture conditions

Human GC cell lines (AGS and HGC-27) were obtained from the Shanghai Institute of Biochemistry and Cell Biology, Chinese Academy of Sciences (Shanghai, China). Cancer cells were cultured with RPMI 1640 medium (Invitrogen, Grand Island, NY, USA) supplemented with 10% FBS at 37°C in a humid atmosphere with 5% CO_2_.

### Cell transfection

The lentiviral hsa_circ_0005927 overexpression vector (LV5-Circ_5927) and lentiviral hsa_circ_0005927 silencing vector (LV3-Circ_5927) were designed and constructed by GenePharma Co., Ltd. (Shanghai, China). When the AGS and HGC-27 cells had reached approximately 40-60% confluence, they were infected with 1×10^7^ recombinant lentivirus transducing units, followed by treatment with 0.75 μg/mL puromycin for one week.

### Real-time analysis of cell proliferation

Cell proliferation was analyzed with a real-time cell analyzer (RTCA; ACEA Biosciences, San Diego, CA, USA). RPMI 1640 medium was added to each well; and the plates were incubated at 37°C for 5 min for background measurement. GC cells were seeded in 96-well E-plates at a density of 5000 cells/well. Stationary cultivation was performed to equilibrate the E-plate for 30 min; then the E-plate was placed in the RTCA Analyzer and the impedance readings began.

### Plate colony formation assay

GC cells were seeded into 6-well plates at a density of 500 cells/well for plate colony formation. Approximately 2 weeks later, the colonies were fixed with 4% paraformaldehyde (Solarbio, Beijing, China) for 10 min and then stained with 0.1% crystal violet staining solution (Solarbio). The experiments were performed in triplicate.

### Transwell migration and invasion assays

Cell migration and invasion were assessed using Transwell chambers (Corning, NY, USA). The cells were diluted to 1~1.5×10^5^/mL with serum-free RPMI 1640 medium. Two hundred microlitres of cell suspension was added to the upper Transwell chamber, while 600 μL of RPMI 1640 medium supplemented with 20% FBS was added to the bottom chamber. After 24 h incubation, the cells migrating from the upper chamber were subjected to 0.1% crystal violet staining and then observed under an electron microscope. Four randomly selected fields per well were analyzed. The experiment was repeated three times. For the invasion assays, the upper chamber was pretreated with 100 µg/mL Matrigel (Solarbio).

### Nucleoplasmic separation

A PARIS™ Kit (Ambion) was used to separate the nuclear and cytoplasmic fractions prior to RNA isolation. The experimental steps and conditions followed the manufacturer's instructions.

### Transcriptome sequencing

Transcriptome sequencing was performed on an Illumina NovaSeq 6000 platform, and 150 bp paired-end reads were generated. Raw reads in FASTQ format were first processed using fastp, and the low-quality reads were removed to obtain clean reads. Then, approximately 3000 clean reads for each sample were retained for subsequent analyses. Differential expression analysis was performed using DESeq2. A *q* value<0.05 and |log2FC|>1.0 were set as the thresholds for significant differentially expressed genes (DEGs). Transcriptome sequencing and analysis were conducted by OE Biotech Co., Ltd. (Shanghai, China).

### Immune infiltration analysis

Relationships between the expression level of key target genes and immune cell infiltrations were analysed by R packages ggplot2[3.3.6] based on estimate[1.0.13] and cibersort algorithm.

### Statistical analysis

Statistical analyses were performed by Statistical Product and Service Solutions (SPSS) 19.0 software or GraphPad (version 8.02). Nomogram prediction models and figures were analyzed and generated with the R software package “rms[6.4.0]”. Bioinformatic analysis was performed using the OECloud tools at https://cloud.oebiotech.com/task/. *P*<0.05 was considered to indicate statistical significance.

## Results

### Hsa_circ_0005927 is downregulated during gastric carcinogenesis

**Figure 1:**
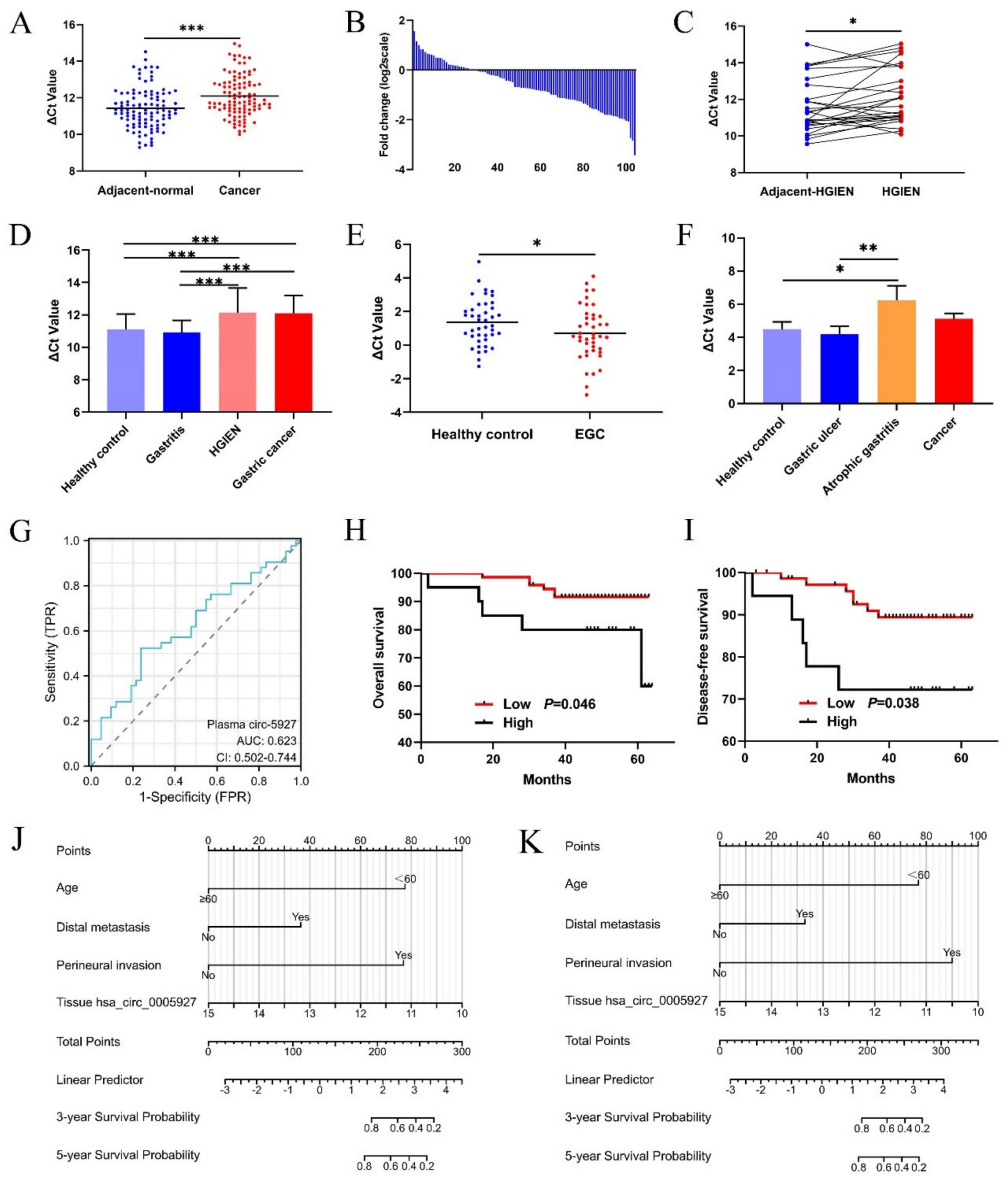
** Clinical significance of hsa_circ_0005927 in gastric cancer.** (A) Expression levels of hsa_circ_0005927 in gastric cancer tissue and adjacent normal tissue (*n*=104). (B) Hsa_circ_0005927 was significantly downregulated in 75% (78/104) of gastric cancer tissues. (C) Hsa_circ_0005927 in high-grade intraepithelial neoplasia (HGIEN) tissues of the stomach (*n*=27). (D) Hsa_circ_0005927 expression levels in various stages of gastric carcinogenesis: healthy control group (*n*=37), gastritis group (*n*=34), high-grade gastric intraepithelial neoplasia group (*n*=27), and gastric cancer group (*n*=104). (E) The plasma Hsa_circ_0005927 level was significantly increased in the early gastric cancer group. (F) Hsa_circ_0005927 levels in gastric juice: healthy control group (*n*=42), gastric ulcers (*n*=30), atrophic gastritis (*n*=16) and gastric cancer (*n*=30). (G) ROC curves of plasma hsa_circ_0005927. (H) Kaplan-Meier survival plot of overall survival. (I) Kaplan-Meier survival plot of disease-free survival. (J) Nomogram prediction models for predicting overall survival in gastric cancer patients (C-index, 0.855). (K) Column chart showing the nomogram for predicting disease-free survival in gastric cancer patients (C-index, 0.855). (*P<0.05, **P<0.01, ***P<0.001).

Compared with that in paired nontumorous tissues, hsa_circ_0005927 expression was significantly decreased in 75% (78/104) of GC tissues (Fig. 1A and Fig. 1B). As observed in GC samples, hsa_circ_0005927 expression also showed a significant decreasing trend in gastric high-grade intraepithelial neoplasia (HGIEN) tissues, which are considered precancerous lesions of GC (Fig. 1C). Moreover, consistent with the above results, hsa_circ_0005927 expression was downregulated only in HGIEN tissues and GC tissues from various stages of gastric carcinogenesis (Fig. 1D). No significant differences were found between the HGIEN group and the GC group (Fig. 1D). Thus, abnormal expression of hsa_circ_0005927 is strongly correlated with GC.

### Relationships between tissue hsa_circ_0005927 levels and clinicopathological factors in GC patients

As shown in Table 1, hsa_circ_0005927 expression in GC tissue was closely associated with lymphatic metastasis and distal metastasis. Patients in the group with low hsa_circ_0005927 expression had a lower risk of developing lymphatic node and distant metastasis (Table 1). Interestingly, Kaplan-Meier analysis revealed that GC patients in the low hsa_circ_0005927 expression group had longer overall survival (OS) and disease-free survival (DFS) than did those in the high hsa_circ_0005927 expression group (Fig. 1H and Fig. 1I).

### Clinical diagnostic value of hsa_circ_0005927 expression in plasma and gastric juice

The plasma and gastric juice RT-qPCR products were sequenced to confirm the presence of hsa_circ_0005927 (Supplementary Fig. 1). Then, plasma hsa_circ_0005927 expression in the healthy group and EGC group was measured by qRT-PCR. As shown in Figure 1E, plasma hsa_circ_0005927 levels were significantly increased in the EGC group, in contrast to its expression in GC tissues. However, in gastric juice, no significant changes in hsa_circ_0005927 levels were found in the cancer group, but hsa_circ_0005927 levels were slightly lower in the atrophic gastritis group than in the healthy control group (Fig. 1F).

A ROC curve was constructed to investigate the potential value of plasma hsa_circ_0005927 expression in EGC screening. When the cutoff value was 0.535, the sensitivity and specificity were 52.38% and 76.19%, respectively. The area under the ROC curve (AUC) reached 0.623 (95% confidence interval [CI], 0.502-0.744; *P*<0.001; Fig. 1G). Moreover, plasma carcinoembryonic antigen (CEA) level slightly increased in 4.35% (2/46) of patients with EGC when its cut-off value was 5ng/mL. Compared with plasma CEA, plasma hsa_circ_0005927 is superior to CEA in terms of sensitivity.

### Construction and validation of the prognostic nomogram model

Univariate Cox regression analysis revealed that clinicopathological parameters such as age, lymphatic metastasis, distal metastasis, venous invasion, and perineural invasion were potential risk factors associated with OS and DFS (Supplementary Table 1). Then, based on the above results, multivariate Cox regression analysis further selected independent variables to establish a multivariate regression model (Supplementary Table 1). Positive risk factors such as age, distal metastasis, and perineural invasion were ultimately selected and combined with tissue hsa_circ_0005927 expression; all of these factors were incorporated into prognostic nomogram prediction models. The nomograms showed good accuracy in predicting OS (C-index, 0.855; Fig. 1J) and DFS (C-index, 0.855; Fig. 1K). The calibration curves indicated that the models had good discriminative ability for prognostic prediction (Supplementary Fig. 2).

### Effects of hsa_circ_0005927 on cell proliferation, invasion and metastasis

**Figure 2:**
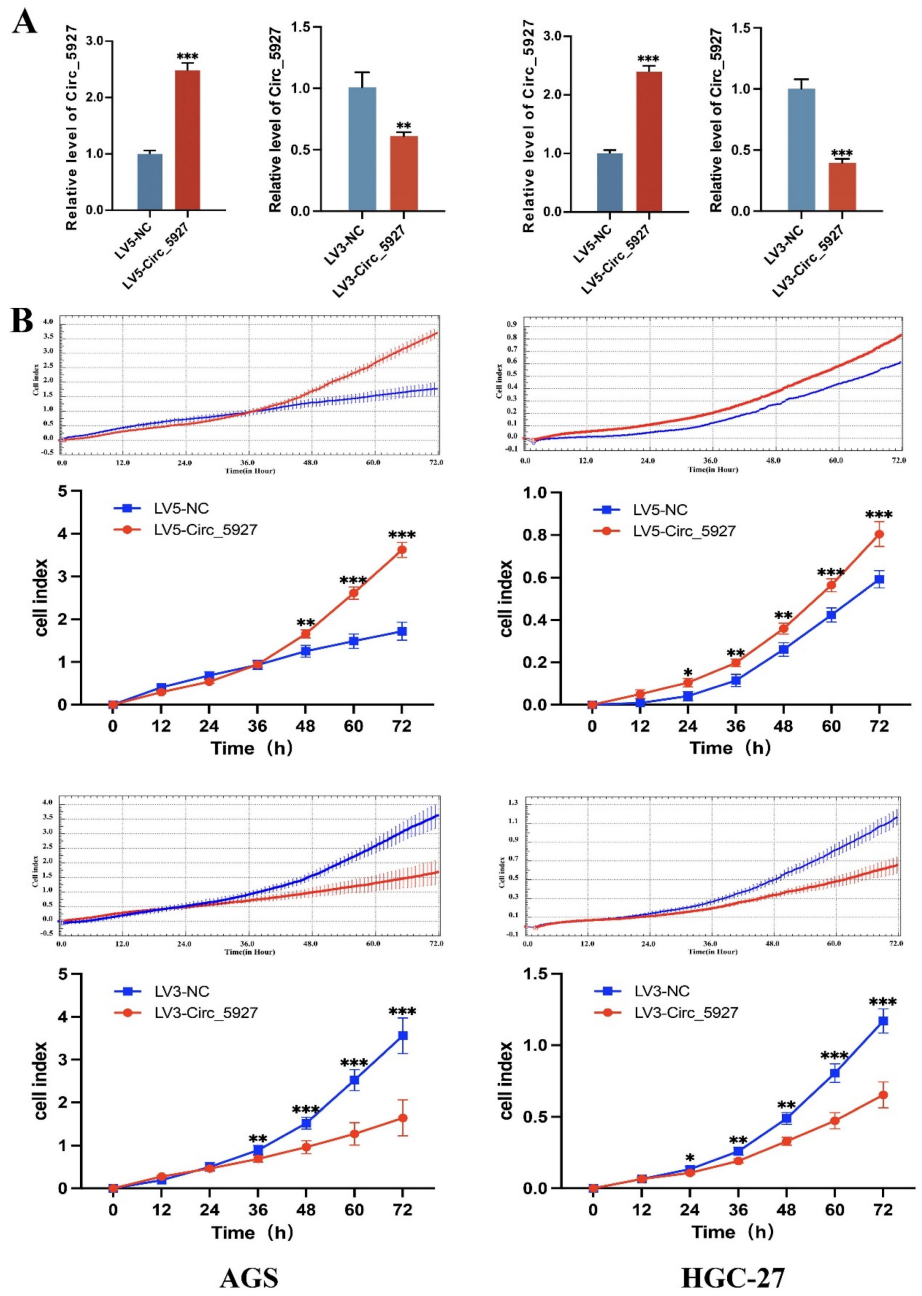
** Overexpression and silencing of hsa_circ_0005927 in gastric cancer cells.** (A) A lentiviral hsa_circ_0005927 overexpression vector (LV5-Circ_5927) and a lentiviral hsa_circ_0005927 silencing vector (LV3-Circ_5927) were constructed and used to regulate the expression level of hsa_circ_0005927 in AGS and HGC-27 cells. (B) Detection of cell proliferation by RTCA. Overexpression of hsa_circ_0005927 promoted AGS and HGC-27 cell proliferation, while silencing of hsa_circ_0005927 significantly inhibited cell growth. The data are presented as the means ± SDs. (*P<0.05, **P<0.01, ***P<0.001).

**Figure 3:**
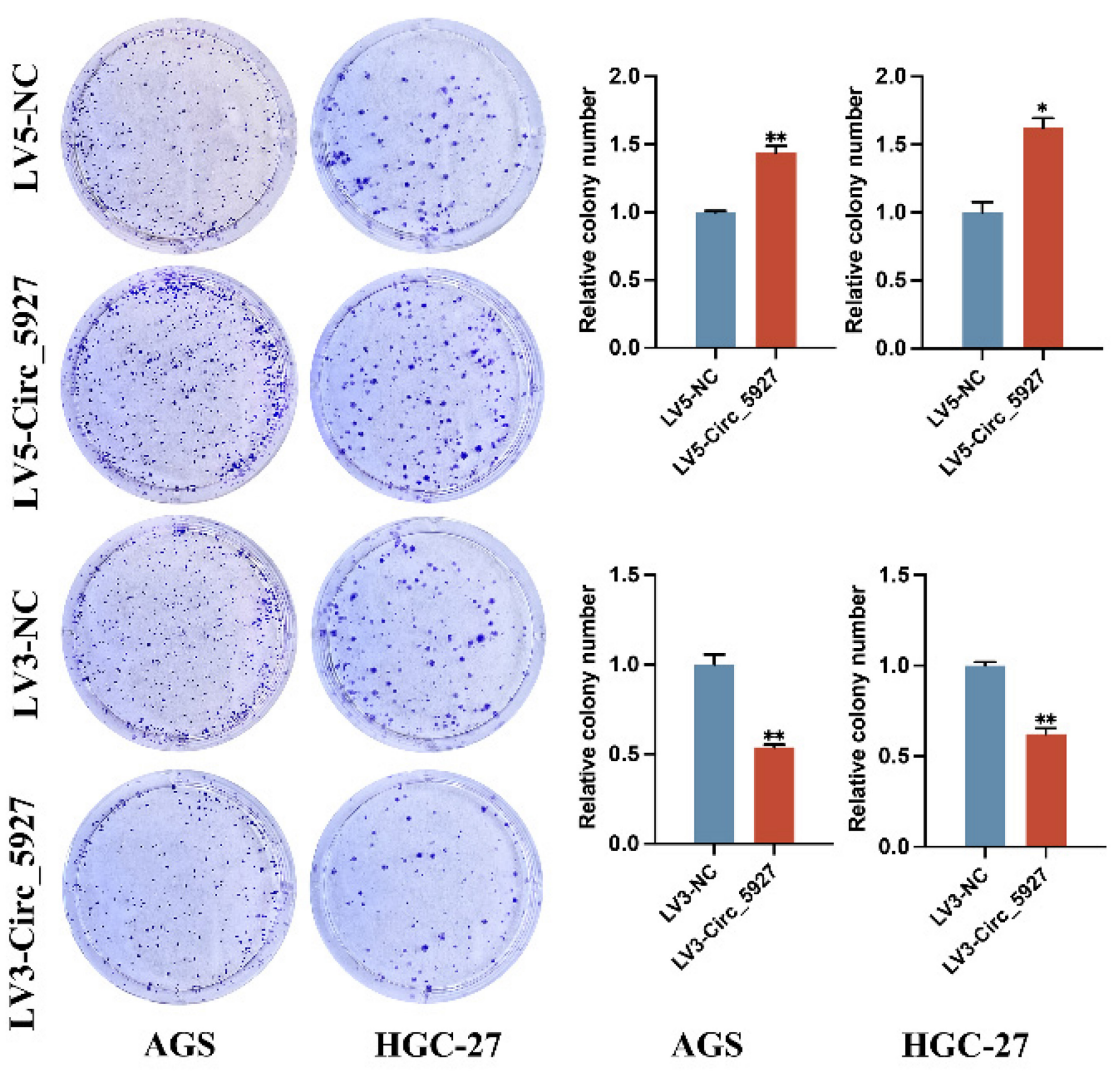
** Plate colony formation assay.** Colony formation was promoted in AGS and HGC-27 cells transfected with LV5-circ_5927 and LV3-circ_5927. The data are presented as the means ± SDs. (*P<0.05, **P<0.01).

A lentiviral hsa_circ_0005927 overexpression vector (LV5-Circ_5927) and a lentiviral hsa_circ_0005927 silencing vector (LV3-Circ_5927) were constructed and used to regulate the expression level of hsa_circ_0005927 in AGS and HGC-27 cells. RT-qPCR results demonstrated that overexpression of hsa_circ_0005927 significantly elevated the expression level of hsa_circ_0005927 in AGS and HGC-27 cells, whereas silencing of hsa_circ_0005927 significantly decreased the expression level of hsa_circ_0005927 (Fig. 2A). Subsequently, RTCA showed that overexpression of hsa_circ_0005927 promoted AGS and HGC-27 cell proliferation, while silencing of hsa_circ_0005927 significantly inhibited cell growth (Fig. 2B). Similarly, colony-forming ability was significantly increased after overexpressing hsa_circ_0005927 in AGS and HGC-27 cells but significantly decreased when hsa_circ_0005927 expression was silenced (Fig. 3).

**Figure 4:**
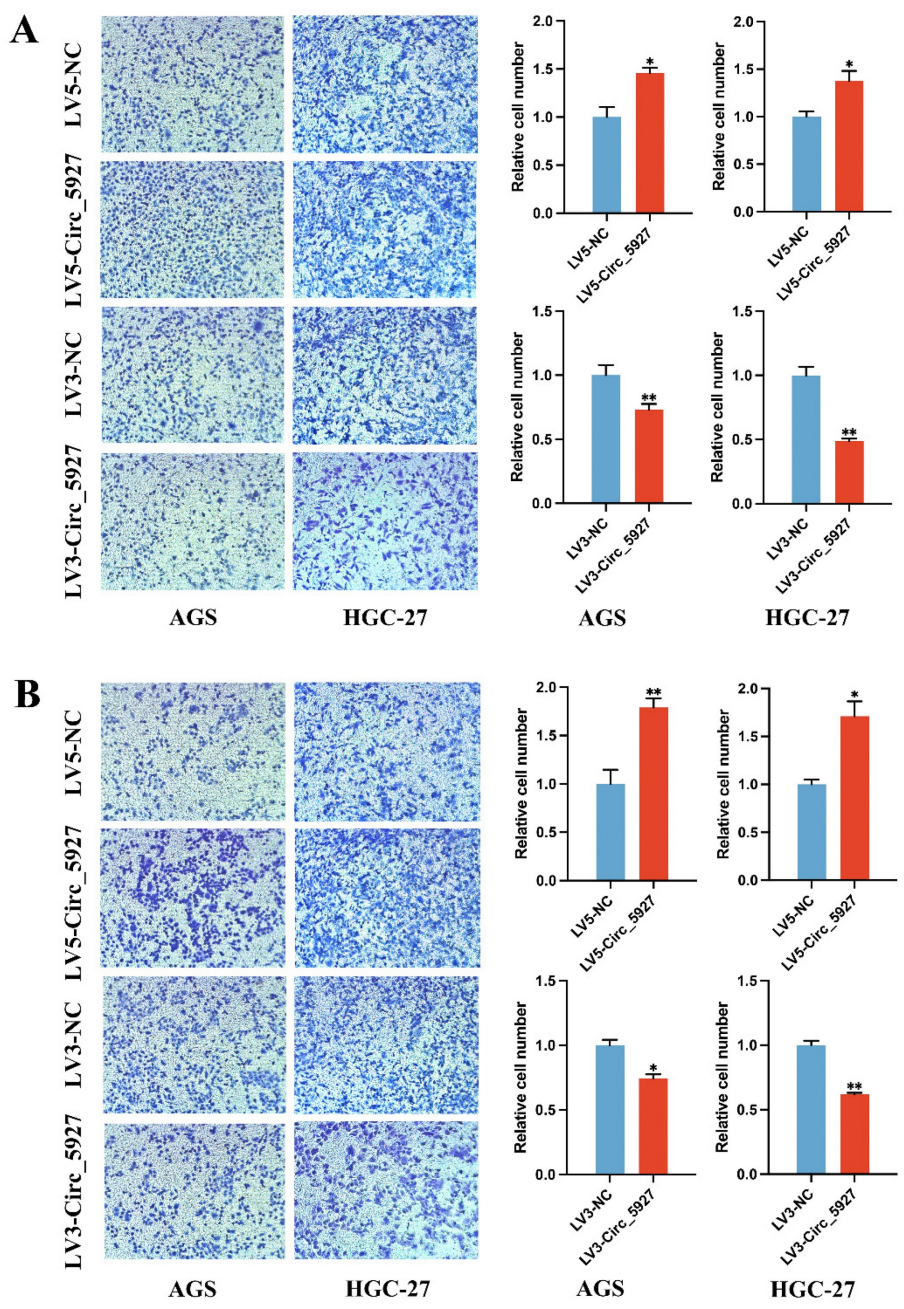
** Transwell migration and invasion assays.** Overexpression of hsa_circ_0005927 significantly promoted the migration (A) and invasion (B) of AGS and HGC-27 cells. Conversely, silencing hsa_circ_0005927 significantly inhibited the migration and invasion of these gastric cancer cell lines. The data are presented as the means ± SDs. (*P<0.05, **P<0.01).

Moreover, Transwell assays confirmed that overexpression of hsa_circ_0005927 significantly promoted the migration (Fig. 4A) and invasion (Fig. 4B) of AGS and HGC-27 cells. Conversely, silencing hsa_circ_0005927 significantly inhibited the migration and invasion of these GC cell lines (Fig. 4).

### Annotation of hsa_circ_0005927 function in gastric carcinogenesis and metastasis from an RNomics perspective

**Figure 5:**
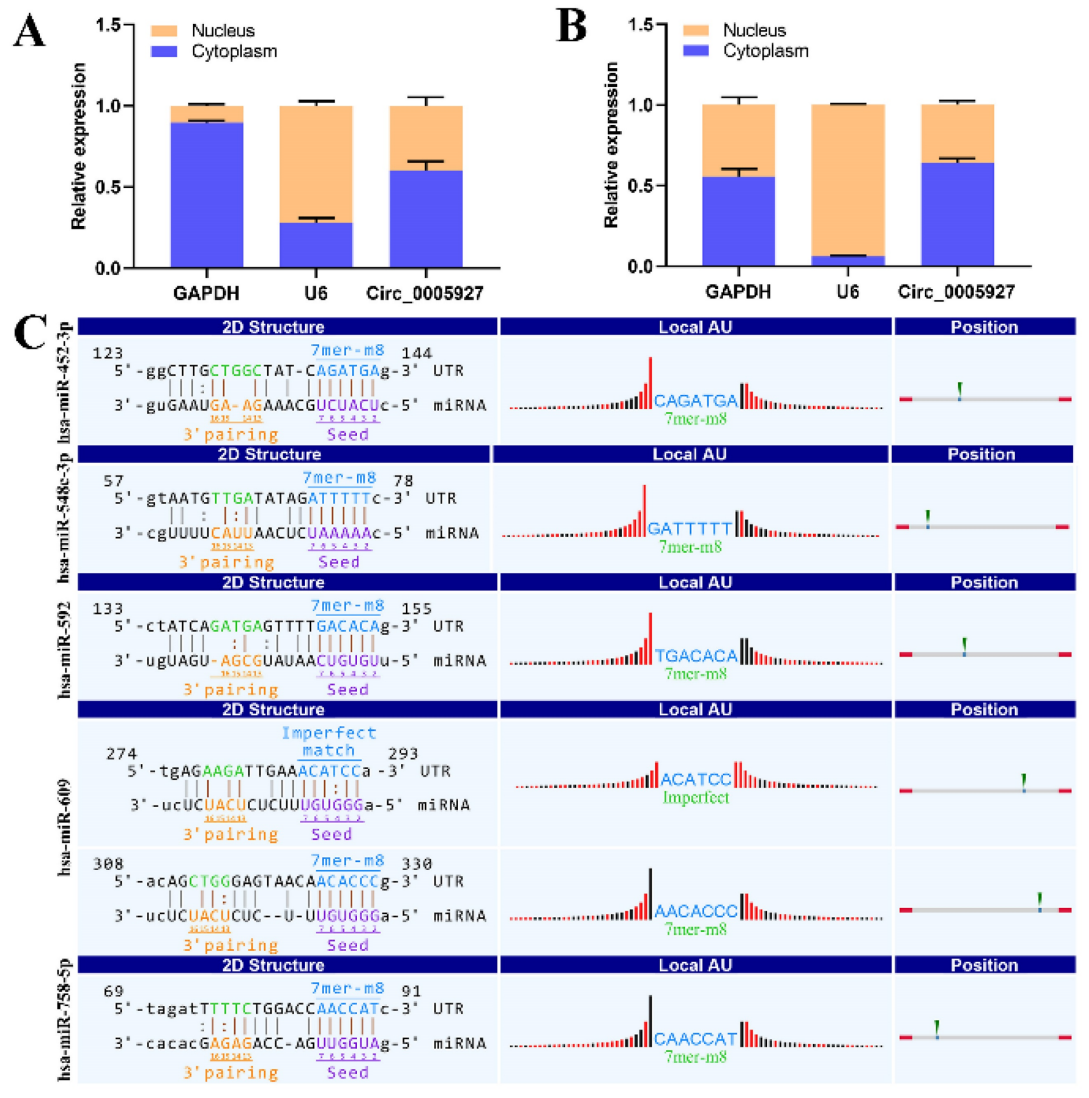
** Nucleoplasmic separation and miRNA prediction.** (A) Nucleoplasmic separation confirmed that hsa_circ_0005927 is located mainly in the cytoplasm of AGS cells. (B) Nucleoplasmic separation confirmed that hsa_circ_0005927 is located mainly in the cytoplasm of HGC-27 cells. (C) Prediction of miRNA interactions.

Nucleoplasmic separation experiments were performed to confirm the distribution of hsa_circ_0005927 in GC cells. As expected, the results demonstrated that hsa_circ_0005927 was mainly distributed in the cytoplasm of GC cells (Fig. 5A-B). miRNA target prediction with TargetScan and miRanda software revealed that hsa_circ_0005927 has the potential to act as a miRNA sponge, and structurally, it possesses multiple miRNA response elements (MREs) that interact with hsa-miR-452-3p, hsa-miR-548c-3p, hsa-miR-592, hsa-miR-609, and hsa-miR-758-5p, which subsequently play biological roles in regulating gene expression in the cytoplasm (Fig. 5C).

**Figure 6:**
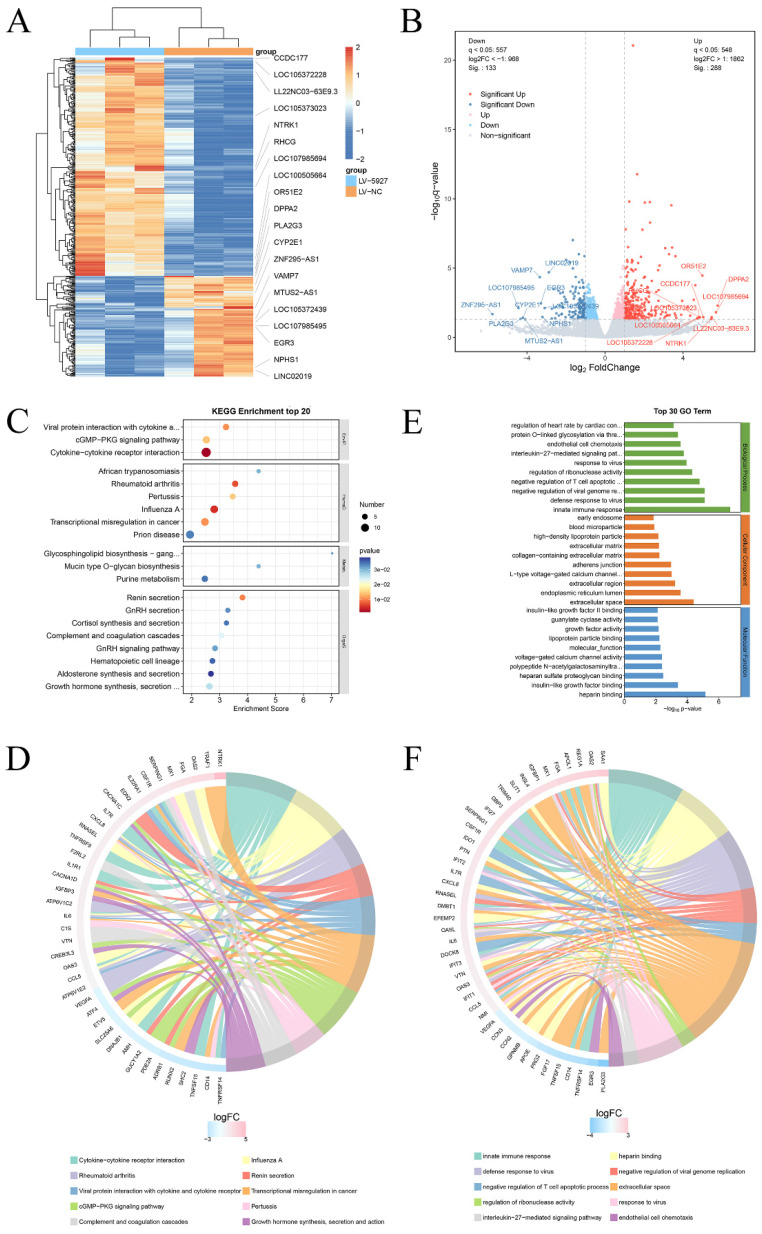
** Annotation of hsa_circ_0005927 functions in gastric carcinogenesis and metastasis.** (A) Hierarchical cluster analysis of downstream target genes after overexpression of hsa_circ_0005927 in AGS cells. (B) Volcano plots were generated to visualize the differential expression of the target genes. (C) KEGG analysis of target genes. (D) KEGG enrichment analysis chord diagram showing the 10 classifications with the smallest q values or p values and their relationships with the corresponding genes. (E) GO analysis of target genes. (F) GO enrichment analysis chord diagram showing the 10 classifications with the smallest q-values or p values and their relationships with the corresponding genes.

Transcriptome sequencing was subsequently performed after overexpression of hsa_circ_0005927 in GC cells (Fig. 6A). A total of 421 downstream target genes with significant changes in mRNA levels were detected, of which 288 were upregulated and 133 were downregulated (Fig. 6B). However, the expression level of host gene VDAC3 remains unchanged. GO analysis revealed that the downstream targets of hsa_circ_0005927 were involved in a variety of biological functions and signaling pathways, such as negative regulation of the T-cell apoptotic process, the interleukin-27-mediated signaling pathway, growth factor activity, and guanylate cyclase activity (Fig. 6C). KEGG analysis revealed that the main biological roles of hsa_circ_0005927 downstream targets were related to transcriptional misregulation in cancer, the cGMP-PKG signaling pathway, the GnRH signaling pathway, etc. (Fig. 6E). Enrichment analysis and chordal plots further displayed the 10 classifications with the smallest q-values or *p*-values and their relationships with the corresponding genes (Fig. 6D-F).

**Figure 7:**
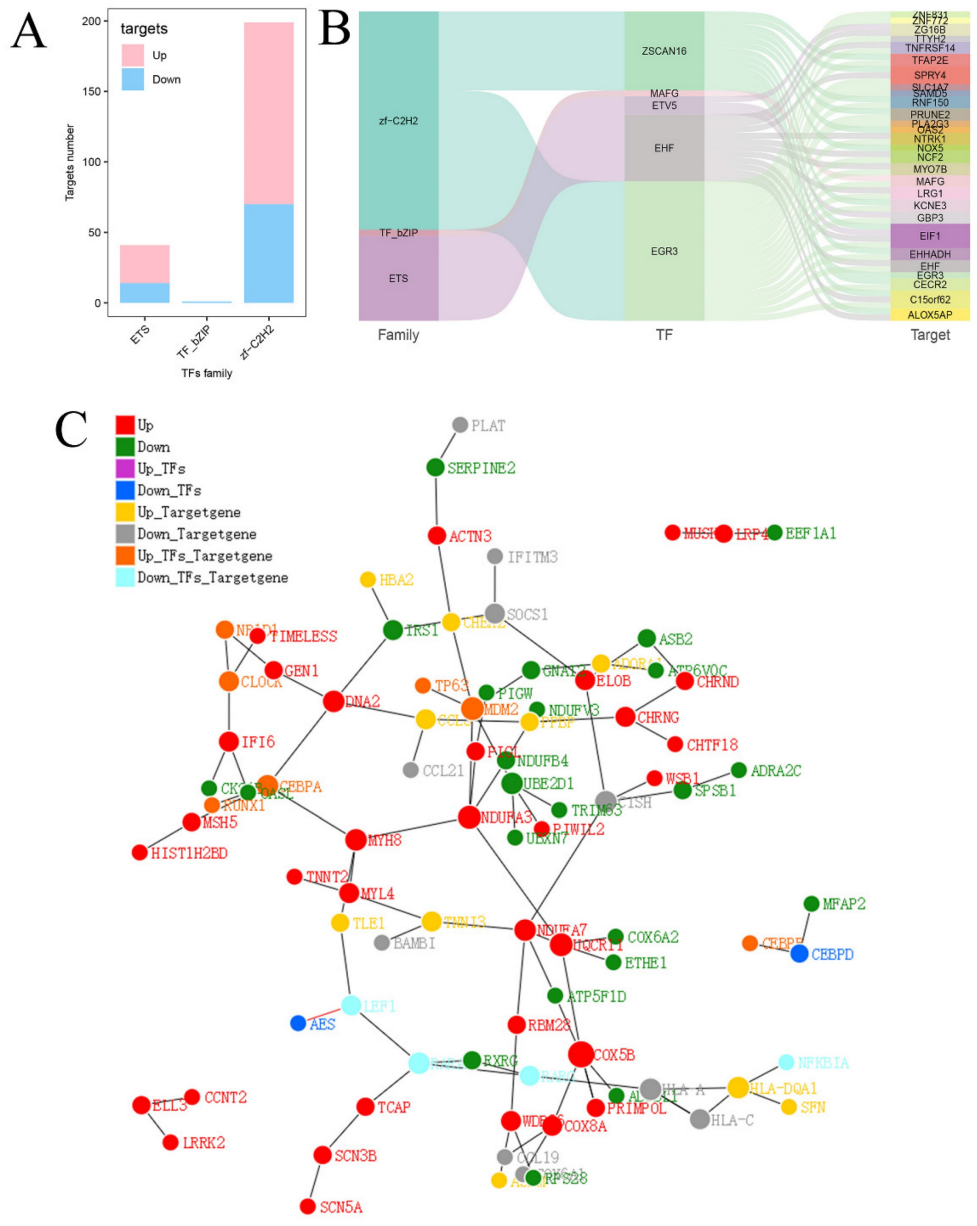
** Analysis of predicted transcription factors and downstream target genes of hsa_circ_0005927.** (A) Relationships between transcription factor families and differentially expressed target genes. (B) Sankey diagram showing the relationships among transcription factor families, differential transcription factors, and target genes. (C) Protein-protein interaction network analysis plot displaying the relationships among transcription factor families, differential transcription factors, and target genes.

Finally, the relationships between transcription factor families and DEGs were further investigated. Twenty-seven upregulated target genes and 14 downregulated target genes were identified in the ETS family, 1 downregulated target gene was identified in the TF_bZIP family, and 129 upregulated target genes and 70 downregulated target genes were identified in the zf-C2H2 family (Fig. 7A). Three major transcription factor families as well as the connections between differential expressed transcription factors and downstream target genes are depicted in Figure 7B. A protein-protein interaction (PPI) network diagram including the transcription factors and their downstream targets was constructed to show their interrelationships (Fig. 7C).

### Immune infiltration landscape of key target genes

**Figure 8:**
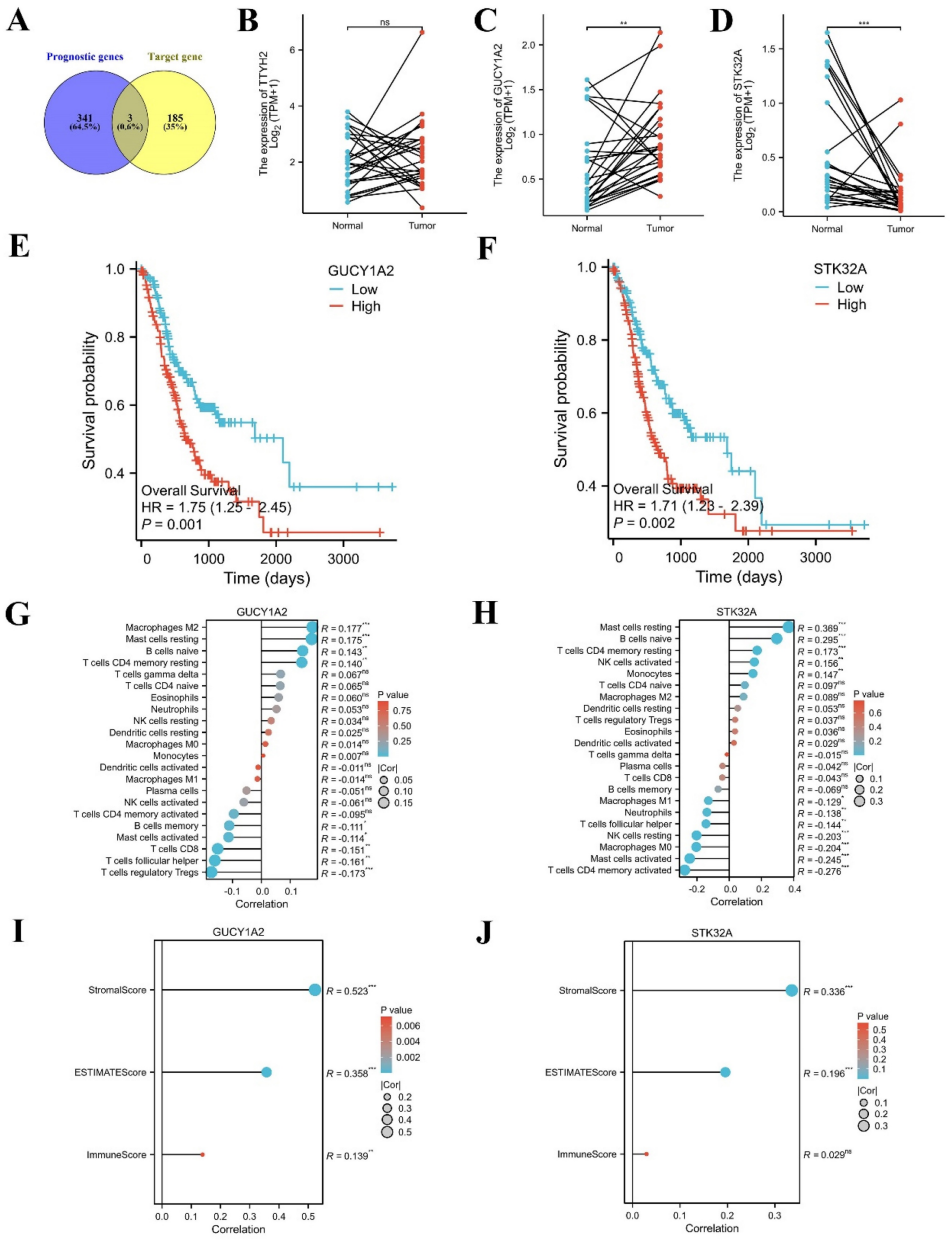
**Immune infiltration landscape of key target genes.** (A) Potential key target genes were obtained by intersection of prognostic genes and target genes (|log2FC|≥1.5). (B-D) The differential expression of potential key target genes were verified between paired normal and tumor samples. (E-F) GUCY1A2 and STK32A were confirmed as key target genes by Kaplan-Meier analysis. (G-H) The lollipop diagram of the expression of GUCY1A2, STK32A and immune cell infiltration. (I-J) The stromal score, estimate score, immune score of different expression level of GUCY1A2, STK32A in TCGA cohort.

TCGA-STAD data were downloaded and sorted out from TCGA database (https://portal.gdc.cancer.gov), and survival [3.3.1] package was used for fitting overall survival regression combined with the clinical data of GC patients. 344 prognostic related genes with hazard ratio (HR) greater than 1.6 were found. Potential key target genes TTYH2, GUCY1A2, and STK32A were obtained by intersection of prognostic genes and target genes (|log_2_FC|≥1.5) (Fig. 8A). Then, based on public data, we directly verified the differences between paired normal and tumor samples and found that GUCY1A2 and STK32A were significantly differentially expressed in GC (Fig. 8B-D). Kaplan-Meier analysis confirmed that higher GUCY1A2 and STK32A expression accompanied with shorter overall survival time (Fig. 8E, F). Moreover, relationship between key target genes GUCY1A2 and STK32A and immune cell infiltration in GC microenvironment were investigate. Our results showed that GUCY1A2 expression was significantly associated with the infiltration of immune cells such as macrophages M2, mast cells resting and B cells naive (Fig. 8G), whereas STK32A expression was significantly associated with immune cells such as mast cells resting, B cells naive, T cells CD4 memory resting and NK cells activated (Fig. 8H). Finally, both GUCY1A2 and STK32A were related to the ESTIMATE score and stromal score (Fig. 8I, J).

## Discussion

CircRNAs are a class of endogenous circular RNA molecules that are produced via variable splicing of precursor RNA and are bound via reverse covalent bonds connecting the 5'- and 3'-ends 10, 11. An increasing number of studies have demonstrated that circRNAs have multiple important biological functions 12. Structurally, circRNAs possess MREs, which act as a miRNA sponge by adsorbing different specific miRNAs to regulate the expression of downstream targets via complementary base pairing 13. In addition, circRNAs can interact with other types of RNAs or proteins to regulate gene expression at the transcriptional and posttranscriptional levels; and a small portion of circRNAs even have protein translation functions 14, 15.

Functioning as competitive endogenous RNAs (ceRNAs) is the main mechanism by which circRNAs regulate gene expression 16. The term ceRNA refers to competitive binding of miRNAs to endogenous RNA molecules with the same MREs to regulate each other's expression levels 17. CircRNAs are rich in MREs, which can act as miRNA sponges to adsorb specific miRNAs and prevent miRNAs from binding to downstream RNA targets in a complementary base pairing manner, thus regulating the expression levels of target genes 18.

Recent studies have revealed that circRNAs interact with disease-related miRNAs and participate in the occurrence and development of GC through the ceRNA network, which is closely related to patient prognosis. Abnormally high levels of hsa_circ_0092306 in GC tissue allow hsa_circ_0092306 to act as a miRNA sponge to adsorb miR-197-3p and prevent miR-197-3p from binding to its downstream target oncogene PRKCB, which ultimately inhibits cell apoptosis and promotes cancer cell migration and invasion 19. The expression level of hsa_circ_0092306 in GC is positively correlated with patient tumor size, histological grade, TNM stage, and lymph node metastasis 19. In another study, Liang et al*.* reported that low expression of circRAB31 in GC is closely associated with multiple prognostic factors, such as tumor size, cell differentiation, and lymph node metastasis 20. Mechanistic research further revealed that circRAB31 acts as a miR-885-5p sponge to indirectly downregulate the expression of the tumor suppressor gene PTEN, inhibit the PI3K/AKT pathway by regulating AKT phosphorylation, and promote the proliferation, migration, and invasion of GC cells 20. Moreover, in the field of GC, more circRNAs, such as circREPS2, circEVI5, circCYFIP2, and hsa_circ_0000199, have been shown to indirectly regulate the expression levels of target genes and alter the biological behavior of GC cells by adsorbing their specific miRNAs through a ceRNA mechanism 21-24. Therefore, functioning as ceRNAs is an important mechanism by which circRNAs regulate gene expression, and the study of related ceRNAs will provide new ideas for combatting the pathogenesis of GC.

In this study, we found that hsa_circ_0005927 was downregulated only in HGIEN tissues and GC tissues during gastric carcinogenesis (Fig. 1D), which indicates that its expression is strongly correlated with GC. Clinicopathological factor analysis suggested that hsa_circ_0005927 expression in GC tissue was correlated not only with lymphatic metastasis and distal metastasis but also with OS and DFS (Table 1). Interestingly, hsa_circ_0005927 also exists in plasma and gastric juice and can be detected by RT-qPCR. As a screening biomarker for GC, plasma hsa_circ_0005927 showed significantly increased expression in the early stages of GC, with a sensitivity and specificity of 52.38% and 76.19%, respectively (Fig. 1E). Our results indicate that hsa_circ_0005927 has the potential to serve as a screening and prognostic biomarker for GC.

Subsequently, cell experiments confirmed that overexpression of hsa_circ_0005927 promoted the proliferation, migration, and invasion of the GC cells. In contrast, silencing hsa_circ_0005927 expression exerted significant inhibitory effects (Fig. 2-4). The results of cell experiments and tissue pathological factor analysis indicated that hsa_circ_0005927 plays an important role as an oncogene in gastric carcinogenesis and metastasis. Moreover, nucleoplasmic separation experiments suggested that hsa_circ_0005927 was mainly distributed in the cytoplasm (Fig. 5A). Based on the distribution of hsa_circ_0005927 in cancer cells, we speculate that miRNA sponges may be the main way for hsa_circ_0005927 to exert biological function. miRNA target prediction revealed that hsa_circ_0005927 had the potential to act as a miRNA sponge, and structurally, it possessed multiple MREs that interact with five miRNAs and regulated gene expression via ceRNA effects (Fig. 5C). Transcriptome sequencing after overexpression of hsa_circ_0005927 in GC cells revealed that a total of 421 downstream target genes were significantly differentially expressed (Fig. 6B). Finally, GO and KEGG analyses revealed that the functions of these target genes were mainly involved in the negative regulation of the T-cell apoptotic process, the interleukin-27-mediated signaling pathway, growth factor activity, guanylate cyclase activity, transcriptional misregulation in cancer, the cGMP-PKG signaling pathway, and the GnRH signaling pathway during gastric carcinogenesis and metastasis (Fig. C-F). Potential key target genes GUCY1A2 and STK32A were obtained by intersection of prognostic genes and target genes (|log2FC|≥1.5) (Fig. 8A). GUCY1A2 expression was significantly associated with the infiltration of immune cells such as macrophages M2, mast cells resting and B cells naive (Fig. 8G), whereas STK32A expression was significantly associated with immune cells such as mast cells resting, B cells naive, T cells CD4 memory resting and NK cells activated (Fig. 8H). Both GUCY1A2 and STK32A were related to the ESTIMATE score and stromal score (Fig. 8I, J). The above results suggest that hsa_circ_0005927 has an important anti-oncogenic role in gastric carcinogenesis and metastasis.

In this study, we explored the biological function and clinical significance of hsa_circ_0005927 in the invasion and metastasis of GC. However, our study also has some potential limitations. On the one hand, our sample size is not large enough, and there is a lack of plasma from patients with non-tumor lesions in the stomach. On the other hand, although we have confirmed that hsa_circ_0005927 mainly exists in the cytoplasm to exert biological effects and found 421 downstream target genes, we were unable to identify a small number of target genes that are affected by other regulatory mechanisms such as binding RNA-binding proteins, acting as protein scaffolds, modulating transcription or epigenetic activities. Therefore, more samples are needed in future studies, and the biological function of hsa_circ_0005927 in cancer cells needs further experimental verification.

## Conclusion

In conclusion, hsa_circ_0005927 was downregulated in HGIEN tissues and GC tissues during gastric carcinogenesis and was associated with proliferation, invasion and metastasis. Our study revealed that hsa_circ_0005927 was a new player related to the invasion and metastasis of GC and is a potential indicator for early GC screening.

## Supplementary Material

Supplementary figures and table.

## Figures and Tables

**Table 1:** Relationship of hsa_circ_0005927 expression levels in cancer tissues with clinicopathological factors of GC patients

Characteristics	No.of case	ΔCt Value	
		Mean±SD	*P* value
Age (y)			
≥60	69	12.011±1.085	0.265
<60	35	12.270±1.163	
Sex			
Male	69	12.167±1.092	0.382
Female	35	11.964±1.158	
Tumor location			
Antrum	52	12.194±1.086	0.052
Angle	8	12.443±1.586	
Body	28	11.629±1.031	
Others	16	12.438±0.880	
Diameter (cm)			
≥5	51	11.958±1.057	0.208
<5	53	12.234±1.158	
Differentiation			
Well	12	12.140±1.053	0.426
Moderate	49	12.235±1.109	
Poor	43	11.931±1.136	
TNM stage			
Early	28	12.183±1.261	0.668
Advanced	76	12.076±1.061	
Borrmann type			
I&II	10	12.406±1.472	0.282
III&IV	66	12.016±0.989	
Invasion			
T_0-2_	39	12.201±1.184	0.469
T_3-4_	65	12.037±1.073	
Lymphatic metastasis			
N_0_	44	12.403±1.202	0.020
N_1-3_	60	11.875±0.995	
Distal metastasis			
M_0_	91	12.188±1.115	0.029
M_1_	13	11.470±0.905	
Venous invasion			
Absent	55	12.146±1.102	0.650
Present	49	12.046±1.134	
Perineural invasion (PNI)			
Absent	47	12.118±1.139	0.874
Present	57	12.083±1.101	
CEA (tissue)			
Positive	94	12.058±1.132	0.257
Negative	10	12.479±0.867	
CA19-9 (tissue)			
Positive	60	12.000±1.113	0.293
Negative	44	12.233±1.111	
