# Supplementary material for: Acting mechanism and clinical significance of hsa_circ_0005927 in the invasion and metastasis of gastric cancer
Source: J Cancer. 2024 May 30;15(13):4081–94. doi: 10.7150/jca.96749 (PMC11212095; doi:10.7150/jca.96749)
Supplement: Supplementary file 1 — Supplementary figures and table. [file jcav15p4081s1.pdf]

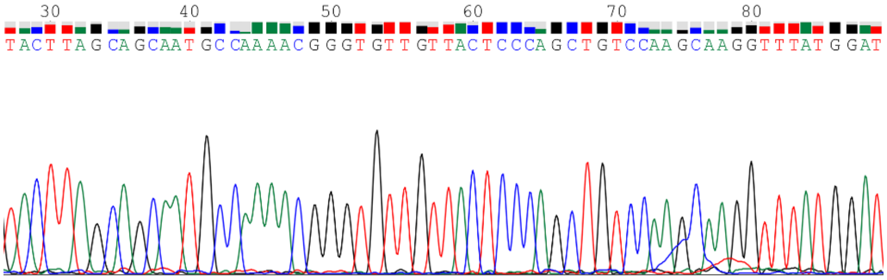

Supplementary figure 1 PCR products were sequenced to confirm the presence of hsa_circ_0005927.

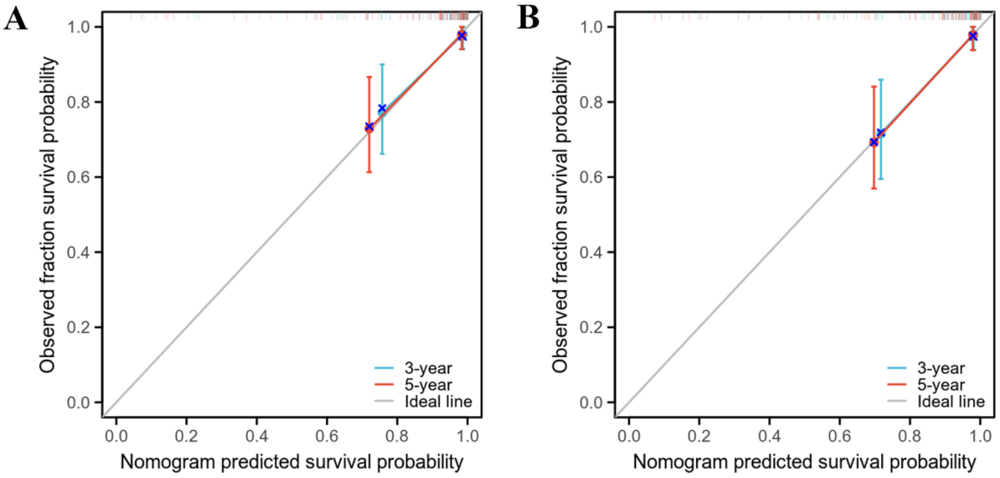

Supplementary figure 2 Calibration curves reflected good discriminative ability of prognosis. (A) OS model. (B) DFS model.

Supplementary table 1 Univariate and multivariate Cox regression analysis of survival time

Characteristics	Univariate analysis		Multivariate analysis	
	Hazard ratio (95% CI)	*P*	Hazard ratio (95% CI)	*P*
Age	0.153 (0.048-0.490)	0.002	0.135 (0.040-0.459)	0.001
Sex	1.327 (0.472-3.731)	0.592		
Diameter	1.126 (0.952-1.331)	0.166		
Differentiation	1.680 (0.738-3.826)	0.216		
Invasion	4.128 (0.891-19.116)	0.070		
Lymphatic metastasis	5.620 (1.268-24.921)	0.023	1.033 (0.172-6.221)	0.971
Distal metastasis	5.756 (2.032-16.301)	0.001	4.047 (1.269-12.905)	0.018
Venous invasion	3.668 (1.164-11.562)	0.026	1.325 (0.354-4.960)	0.676
Perineural invasion	13.441 (1.764-102.381)	0.012	6.980 (0.714-68.210)	0.095
CEA	0.307 (0.085-1.102)	0.070		
CA19-9	0.451 (0.159-1.278)	0.134		
Hsa_circ_0005927	0.607 (0.356-1.034)	0.066		
